# Effects of Iron Overload and Oxidative Damage on the Musculoskeletal System in the Space Environment: Data from Spaceflights and Ground-Based Simulation Models

**DOI:** 10.3390/ijms19092608

**Published:** 2018-09-03

**Authors:** Jiancheng Yang, Gejing Zhang, Dandan Dong, Peng Shang

**Affiliations:** 1 School of Life Sciences, Northwestern Polytechnical University, Xi’an 710072, China; yangjiancheng@mail.nwpu.edu.cn (J.Y.); 2017263324@mail.nwpu.edu.cn (G.Z.); ddd2013@mail.nwpu.edu.cn (D.D.); 2 Key Laboratory for Space Bioscience and Biotechnology, Institute of Special Environment Biophysics, Northwestern Polytechnical University, Xi’an 710072, China; 3 Research & Development Institute in Shenzhen, Northwestern Polytechnical University, Shenzhen 518057, China

**Keywords:** iron overload, oxidative damage, microgravity, radiation, countermeasures

## Abstract

The space environment chiefly includes microgravity and radiation, which seriously threatens the health of astronauts. Bone loss and muscle atrophy are the two most significant changes in mammals after long-term residency in space. In this review, we summarized current understanding of the effects of microgravity and radiation on the musculoskeletal system and discussed the corresponding mechanisms that are related to iron overload and oxidative damage. Furthermore, we enumerated some countermeasures that have a therapeutic potential for bone loss and muscle atrophy through using iron chelators and antioxidants. Future studies for better understanding the mechanism of iron and redox homeostasis imbalance induced by the space environment and developing the countermeasures against iron overload and oxidative damage consequently may facilitate human to travel more safely in space.

## 1. Introduction

With the continued application of the International Space Station, the construction of the Chinese Space Station, the development of rockets and space habitats aimed at sending humans to Mars and other Solar System bodies, the emergence of space travel, and the increase in the participation of private companies in spaceflight activities, humanity is committed to the exploration of the universe. Such missions will be many months, to years long. But these activities offer a number of challenges. When exposed to the spaceflight environment, including microgravity and radiation, there will be variously physiological health alterations, including dysregulation in the immune system [1], dysfunction in the cardiovascular system [2], and disruption in the nervous system [3], etc. Bone loss and muscle atrophy caused by microgravity are also well documented in the human body, and they generally occur in weight-bearing bones and linked muscles, and they need a very long time to recover after going back to earth [4,5]. Due to the absence of countermeasures, these changes can affect the performance and safety of crewmembers seriously during space missions. The current understanding of skeletal and muscular atrophy is the transformation of physical signals in mechanical unloading into molecular signaling processes that induce the loss of calcium and the degradation of myofibrils at the biochemical level, followed by a significant bone and muscle mass loss. However, in addition to the absence of mechanical stimuli, some changes in non-mechanical factors such as iron metabolism and the redox system in humans/animals undergoing the space environment have been reported.

Iron is an essential trace element that plays an important role in human physiology and biochemistry, such as electron transport, oxygen binding, and it acts as a catalyst for hundreds of enzymes [6]. However, iron is a highly transitional metal that catalyzes the formation of reactive oxygen species (ROS) [7]. High-doses of iron dextran-treated iron-overloaded mice led to an increase of ROS and bone resorption, resulting in the disruption of bone structure and material properties, and thereby bone loss [8]. In vitro, excess iron inhibited the proliferation, differentiation, and activity of osteoblasts [9], whereas iron overload promoted osteoclast differentiation and bone resorption activity by accelerating the production of ROS [10]. Similarly, a mouse model of iron overload showed significantly elevated iron content in skeletal muscle, elevated levels of the oxidative stress product malondialdehyde (MDA), decreased muscle mass, reduced mass of fast-twitch muscle fibers and muscle strength, and displayed a low level of exercise ability [11]. Moreover, clinical studies and case reports found that abnormalities of bone and muscle occurred in patients with iron-loading conditions, such as hemodialysis, menopause, and aging [12,13].

Evidence from space sojourns and ground-based analogy models have shown an increase of iron levels and oxidative damage in human/animal, and these increases were closely related to the negative effects of the space environment on the musculoskeletal system. The concentrations of serum ferritin in circulation, an index of iron storage, was increased during/after short- and long-term spaceflights [14,15]. Elevated serum ferritin was positively associated with the increase of oxidative stress markers and the decrease of bone mineral density (BMD) in spacemen during long-term spaceflight on the International Space Station (ISS) [14]. During the head-down bed rest (HDBR) model, a ground-based model for human, volunteers showed that increased iron stores and oxidative stress markers were accompanied by damage to bone and skeletal muscle [16,17]. The rodent hindlimb unloading (HLU) model is a classical mimical for microgravity. Our recent study showed that HLU-induced bone loss in mice was connected with iron overload; we speculated that iron-induced ROS is involved in this process [18]. Indeed, HLU-induced bone loss, at least in part, was regulated by intracellular ROS generation [19]. In HLU-induced muscle atrophy, also showed the phenomenon of enhanced iron level and oxidative damage [20].

In conclusion, these evidences strongly suggest a risky role of iron overload and oxidative damage in the regulation of bone mass and muscle mass in the space special environment. Herein, we summarize the changes in iron metabolism and redox systems in spaceflight and ground-based analogy models, and elaborated on the relationship between these changes and musculoskeletal metabolism. The feasible mechanisms will be discussed as well. Some countermeasures against iron overload and oxidative injuries to prevent bone loss and muscle atrophy will also be included. This review will help researchers to capture the latest research progresses and inspire their possible direction of future studies.

## 2. The Effect of Spaceflight Environment and Its Analogs on Iron Metabolism

### 2.1. Evidence from Spaceflight

Iron plays a crucial role in many biological functions, such as oxygen transport, enzymatic reactions, and energy production. However, excessive levels of iron can damage tissues by catalyzing the generation of ROS that attack cellular DNA, proteins, and membranes [6]. Ferritin, an iron-containing protein, a major iron storage protein, since it can accumulate great amounts of iron [21]. Although there are many indexes available, serum ferritin (SF) concentrations are the most effective indicators to determinate iron status in clinical and public health settings [22]. The observation from short-term (week) and long-term (month) space missions shows an increased SF concentrations in astronauts [16,23]. Recently, a long-term space travel with 23 cosmonauts, the SF concentrations of women and men were increased by about 220% and 70%, respectively, on the 15th day of flight [14]. Moreover, the concentration of soluble transferrin receptors (sTfR) are decreased in spaceflight, suggesting that less iron is being transported [14,23]. These findings have attracted attention, due to excess iron in tissues can damage the metabolism of bone and muscle, destroy immune function, and elevate sensitivity to radiation damage [8,11]. However, although the impact of the spaceflight environment on iron metabolism has been confirmed, the mechanism promoting such regulation remains unexplored. Importantly, we need to know where this increased iron was derived from.

The human body contains 3–5 g of iron, and approximately 65–75% is found in the hemoglobin of red blood cells (RBCs) [24]. Therefore, changes in iron metabolism are closely related to hematological changes in spaceflight. The decrease in RBC mass is a consistent finding after a short-term and long-term duration spaceflight [25,26,27,28,29]. The decline in RBC mass during spaceflight, termed “spaceflight anemia”, was observed as early as the Gemini missions in the 1960s [30]. The decline rate in RBC mass slightly exceeds 1% per day, and the eventual loss is about 10–15% in 10–14 days of spaceflight [15,31]. A explanation to explain the decline in RBC mass is the selective destruction of the youngest circulating RBCs; this process is termed as “neocytolysis” [32]. One consequence of the destroyed RBCs is the subsequent release of iron from these cells into the iron-storage protein. Another cause is that erythropoiesis is suppressed during space flight [33]. Consistently, the concentration of sTfR in serum is a valuable method for the quantitative assay of marrow erythropoietic activity [34], as it is decreased in spaceflight [14,23]. In addition, the elevation of iron stores in spaceflight may be associated with excessive amounts of dietary iron content in the food system of the International Space Station (ISS), since the food in the ISS menu are fortified with iron [35].

### 2.2. Evidence from Ground-Based Analog Models for Spaceflight

Data on the physiological response to the real microgravity are limited, due to spaceflights being infrequent and costly. Hence ground-based models of microgravity were established, including a head-down bed rest (HDBR) model for humans and a hindlimb unloading (HLU) model for animals [36,37].

The model of HDBR, which has been used as a model of spaceflight, is associated with cephalic fluid shift. Unlike spaceflight results, although RBC mass decreased during bed rest [38,39], no changes were observed in SF. Nonetheless, a decreased concentration of serum TfR was found in a long-duration HDBR [16]. These changes suggested that the reduction in RBC mass in HDBR is due to a decrease in erythropoiesis, rather than an increase in erythrocyte destruction. Indeed, on the one hand, a statistically significant suppression of erythropoietin levels, a glycoprotein cytokine, which stimulates erythropoiesis in the bone marrow, occurred while on HDBR [40,41]. On the other hand, there was no change in the indicators of hemolysis mean and fecal urobilinogen concentration at 10 and 21 days of bed rest, compared to the baseline [39]. No significant changes in hematology, iron status, or endocrinology occurred during a 30-day HDBR [42]. Obviously, although bed rest is a valuable analog for spaceflight, it cannot imitate the complexities and uniqueness of spaceflight for all systems.

Due to the limitation of obtaining the experimental sample from human, the rodent HLU model has thus been developed to study the mechanisms of adverse consequences such as bone loss and muscle atrophy that occurs in spaceflight [37]. Similar to the data from spaceflight, increased SF levels were also shown in hindlimb-unloaded mice [18]. Excessive iron is mostly stored into hepatocytes in the liver and reticuloendothelial macrophages in the spleen [43]. Iron deposition in the liver and spleen was significantly increased in mice after unloading for 28 days [18,44]. An increase of the spleen iron content also was found in a study of 7-day HLU rats [45]. Systemic iron metabolism is mainly controlled by hepcidin, a hormone produced by the liver. In iron overload, high hepcidin levels reduces cellular iron export by degrading the iron excretion protein, ferroportin [6]. In agreement with the increased iron stores, the hepcidin level in the liver [44] and serum [18] was significantly higher in mice after HLU for 28 days. Under inflammatory conditions, interleukin-6 (IL-6) and relevant cytokines bind to the IL-6 receptor, leading to the activation of signal transducer and activator of transcription 3 (STAT3), which in turn promotes hepcidin production [6]. After HLU for seven days, the mRNA levels of hepcidin were enhanced in the liver of rats, accompanied by the activation of the IL-6/STAT3 axis [45]. Therefore, these data imply that the changes in iron metabolism may be mediated through upregulating hepcidin via an inflammatory process in microgravity.

## 3. Iron Overload and Its Link to Spaceflight-Induced Oxidative Stress

### 3.1. The Relationship Between Iron and Oxidative Stress

The presence of intracellular iron has a powerful effect on the redox state of the cells at the cellular level, resulting in oxidative stress in individual cells. Iron and its derivatives, such as heme or iron-sulfur (Fe-S) clusters, are essential for the function of ROS-producing enzymes and incorporated into them, including nicotinamide adenine dinucleotide phosphate hydride (NADPH) oxidases, lipoxygenases (LOXs), xanthine oxidase, cytochrome P450 enzymes, and subunits of the mitochondrial electron transport chain [7]. Iron is also found at the active site of the H_2_O_2_-degrading enzyme catalase, found in the peroxisome. As transition elements, iron is prone to participate in a one-electron transfer reaction, capacitating it to accept or release electrons while switching between its ferrous bivalent (Fe^2+^) and ferric trivalent (Fe^3+^) states [46]. ROS, a universal term and a clustering of partially reduced oxygen-containing molecules such as superoxide (O_2_^−^), hydrogen peroxide (H_2_O_2_), and lipid hydroperoxide (ROOH), are known to be involved in several signaling pathways, due to the catalysis of specific cellular redox reactions [7]. However, relatively nontoxic H_2_O_2_ and ROOH are reduced further through the reaction of peroxides, to produce highly reactive hydroxyl (OH^•^) or lipid alkoxy (RO^•^) radicals, which is known as the Fenton reaction, which includes the oxidation of Fe^2+^ to Fe^3+^, and electron transfer to H_2_O_2_ and ROOH [7]. The presence of superoxide further facilitates Fenton chemistry by accelerating the conversion of Fe^3+^ to Fe^2+^, and the complete catalytic electron transport cycle of iron is referred to as the Haber–Weiss reaction [47]. In conclusion, iron-dependent ROS-producing enzymes and labile iron are crucially involved in the formation of ROS. Therefore, iron chelators such as deferoxamine, deferiprone, and deferasirox have been used as treatments for diverse pathologies associated with iron or ROS accumulation [48].

### 3.2. Oxidative Stress in Spaceflight

In normal conditions, there is a dynamic balance between oxidant production and antioxidant defense in the body, termed redox homeostasis. However, spaceflight can cause the imbalance of redox homeostasis in human and animal blood, urine, and tissues [49].

Several studies showed that redox status was disturbed during and/or after a space sojourn in astronauts. The oxidative injury markers, 8-hydroxydeoxyguanosine (8-OHdG) and 8-iso-prostaglandin F2α (8-iso-PGF2α) in urine, were measured during and after spaceflight in the Mir mission crew [50]. The isoprostanes, 8-OHdG and 8-iso-PGF2α, are markers for oxidative damage to DNA and membrane lipids, respectively. Isoprostane excretion was decreased in urine during flight, but it increased post-flight [50]. Similarly, the level of 8-OHdG was unchanged inflight, and enhanced postflight. No changes were observed in the levels of 8-OHdG with bed rest, but isoprostane was elevated during the recovery phase. The changes in isoprostane excretion were due to the decrease of oxygen-free radicals in the electron transport chain, as a result of the reduced energy intake inflight, whereas the increase of oxidative damage markers post-flight were probably caused by a combination of elevated metabolic activity and the loss of some oxidation resistance during spaceflight [50]. The attenuated antioxidant defense is a possible mechanism for the elevated levels of oxidative stress after flight. Antioxidant gene expression, including Mn superoxide dismutase (Mn–SOD), Cu–Zn–SOD, kelch-like ECH-associated protein 1 (KEAP1), glutathione peroxidase 4 (*GPX4*), and the regulator of nuclear factor erythroid 2-related factor 2 (NRF2), were significantly decreased in two hair samples from two spacemen who had flown six months on the ISS [51]. A study assessed the oxidative stress status in 12 Russian cosmonauts, the results exhibited increased granulocyte nitric oxide production and superoxide, increased superoxide dismutase (SOD) activity and glutathione (GSH) oxidation in erythrocyte, and strongly reduced plasma/leucocyte lipophilic antioxidant levels [52]. Recently, a study with 23 crewmembers on missions of 50–247 days, increased SF was positively associated with elevated oxidative stress markers 8-OhdG and 8-iso-PGF2α [14], suggesting that the increase of iron stores may be an inducing factor for oxidative damage.

Coincident with the observations from astronauts, rodents imposed on spaceflight also display changes in redox signaling compared with the ground-based controls. Through the analysis of erythrocytes in mice from a long-term spaceflight, the results showed that the lipid peroxidation products were increased, and antioxidant defenses were induced, with an increase in GSH content compared with ground control erythrocytes [53]. Nonetheless, increased antioxidant defenses were not sufficient to prevent damage from oxidative stress. Female C57BL/6 mice were flown for 13 days on the Space Shuttle Atlantis (STS-135), and skin samples were collected for gene chip assays and metabolic biochemical analysis; the data illustrated that spaceflight induces an alteration in biological and metabolic homeostasis, due to increases in the regulation of ROS production, cellular antioxidants, and tissue remodeling [54]; the liver exhibited a markedly injury reaction to oxidative stress, evidenced through reduced hepatic GSH levels with enhanced ophthalmate, a biomarker for the consumption of GSH [55]; eyes were removed for analysis, and the expression of several genes responsible for regulating ROS production were significantly up-regulated, and the level of hydroxynonenal (HNE) protein, which is an oxidative marker response to lipid peroxidation, was significantly increased in the retina, compared to the ground-control mice [56]. Similarly, male C57BL/6 mice were flown on the STS-135 for 13 days, and one eye sample was fixed for histological sectioning, and 8OHdG staining showed a significantly increased oxidative damage in the eye [57]. In rats, short-term spaceflight (six days) promoted the gene expression of cardiac mitochondrial redox-related enzymes, implying a possible stress response and/or energy metabolism changes [58]. Spaceflight increased the expression of antioxidant genes and markers of oxidative damage in the liver of rats [59]. Furthermore, after an 8-day flight in the STS-63, male rats showed a decrease in the total GSH levels and activities of CuZnSOD, GSH reductase, GSH sulfur-transferase, and catalase in the liver [60].

### 3.3. Oxidative Stress in Ground-Based Models for Spaceflight

Microgravity and space radiation are considered to be major and inevitable risk factors for space sojourn. These environmental stresses, when simulated on the ground, demonstrate an imbalance between oxidant production and antioxidant defense.

Healthy and normal menstruating women were randomly grouped to a bed rest study for 10 days, and oxidative stress markers which advanced oxidation protein products (AOPP) and MDAwere increased, whereas antioxidant status through the measurement of glutathione peroxidase (GPX) was reduced during and after bed rest [61]. Twenty healthy male volunteers participated in a study of HDBR, and serum, salivary MDA, and 8-OHdG levels were significantly elevated after 60 days of analogic microgravity [62]. Moreover, identical results were showed in a 10-day HDBR study [63,64]. These results suggest that oxidative stress in humans is enhanced during bed rest.

In agreement with humans during bed rest, redents that were subjected to HLU in simulated microgravity also exhibit increased oxidative stress. A longer-than-14 d HLU led to MDA levels, ROS, and 8-OHdG concentrations being markedly elevated in the brains of rats [65]. After 21 and seven days of HLU in rats, peroxiredoxin 6 and DJ-1, which resist oxidative damage, were up-regulated in the hippocampus [66]. Mitochondrial regulation of the expression and activity of NADPH oxidases in unloaded rats was involved in the regulation of vascular redox status [67]. HLU resulted in the destruction of muscle antioxidant status, and an enhancement in oxidative stress [68]. Marrow and bone showed markedly elevated intracellular ROS and CuZnSOD in mice with tail-suspension [19]. Tissues obtained from rats after two weeks or three weeks of unloading had significantly increased MDA levels, suggesting that oxidative stress is induced during unloading [69,70,71,72].

Space radiation is another major feature of the space environment, and it is a strong stimulus for oxidative stress [49]. Space-relevant radiation can induce oxidative damage in various tissues of rodents. Lung tissue from mice showed significant oxidative damage, including DNA damage, protein nitrosative stress, and lipid peroxidation after exposure to radiation, similar to space [73,74,75]. Mice exposed to low-doses of ionizing radiation exhibited oxidative damage and apoptosis in the retina [76]. Increased oxidative damage in the heart was displayed in mice exposed to protons (50 cGy) or ^56^Fe (15 cGy) [77]. In high-energy proton-irradiated mouse brains, a significant dose-dependent elevation of ROS and lipid peroxidation, and a reduction of antioxidants were found [78]. Exposure of mice to γ-radiation (^137^Cs, 1–2 Gy) induced the increase of ROS generation and lipid peroxidation in the bone marrow [79]. Similarly with tissues, enhanced oxidative stress also can be found in irradiated cells, such as lung epithelial cells [80], neural precursor cells [81], and neuroblastoma cell lines [82].

In conclusion, evidence from spaceflight and ground-based rodent models, including analogic microgravity and space-relevant radiation, shows that oxidative damage occurs in response to the space environment.

## 4. Iron and ROS Signaling in Spaceflight-Induced-Bone Loss and Muscle Atrophy

### 4.1. Iron and ROS Signaling in Spaceflight-Induced-Bone Loss

Although iron is essential in the human body, iron is a highly transitional metal that catalyzes the formation of hydroxyl radicals. As a result, iron levels that exceed the cell’s tolerance threshold can induce a variety of adverse consequences. Data from in vitro experiments, animal models, and clinical studies demonstrated that excess iron has direct deleterious effects on bone metabolism [12]. Iron is strongly linked to ROS production, due to the Fenton reaction; thus, excess iron-induced bone loss may be through an increase in ROS production. An iron overload mouse established through the injection of iron dextran into C57/BL6 mice for two months, displayed dose-dependent elevated tissue iron levels and ROS, which were accompanied by enhanced bone resorption [8]. In iron-loading rats, the concentrations of SOD in serum was reduced, whereas MDA levels were elevated. Concomitantly, rats showed visible bone abnormalities after iron intervention, such as the calcium content of bone tissue being significantly decreased, the length of the tibia was shortened, the BMD of the femur was decreased, and the damage of the bone microarchitecture was obvious [83]. The bone system is a living tissue in a dynamic balance, and bone remodeling, occurs throughout life to support a healthy skeleton. In bone remodeling, old and damaged bone tissue is removed by osteoclasts, and new bone is produced by osteoblasts [84]. In vitro, iron overload in osteoblasts produced through culturing in a medium supplemented with FAC as a ferric ion donor, and has shown an inhibition of osteoblast biological activity because of increased intracellular iron and ROS levels in a concentration-dependent manner [85]. With regard to osteoclasts, ferric ion-facilitated Receptor Activator of Nuclear Factor kappa B Ligand (RANKL) induced osteoclast formation in both bone marrow-derived macrophages (BMMs) and RAW264.7 cells, and this effect was accompanied by an increase in the levels of ROS and oxidative stress [86]. Therefore, elevated bone dissolution by osteoclasts and reduced bone formation by osteoblasts are conducive to bone loss induced by iron overload.

Bone loss has been considered to be the greatest kind of damage to the health of humans in spaceflight. There is a great deal of data from spaceflight missions that indicate that space causes an imbalance between bone formation and resorption [4]. During flight in astronauts, the concentrations of bone-resorption markers are increased, even when good nutrition and improved physical activity training is implemented [87]. With respect to bone formation, there is only a tendency for bone mass to decrease or not change when comparing pre-flight and post-flight values [88]. However, bone cell culture experiments under aerospace conditions have shown that even under osteogenic induction conditions, spatial microgravity inhibited osteogenic differentiation and induced adipogenic differentiation [89]. In long-term space missions, rates of bone loss in spacemen are about 10 times greater than the data obtained from postmenopausal women [87]. Bone loss in the spaceflight environment is related to iron disorder and oxidative stress. In-flight data showed that the greater the increase in ferritin during flight, the greater the elevation in oxidative stress markers, and the greater the decrease in BMD after long-term spaceflight [14]. Thus, elevated iron stores may be an induction factor for oxidative damage and osteopenia in spaceflight.

Findings from ground-based models of simulated microgravity all demonstrated elevated iron stores, or/and oxidative damage were accompanied by bone loss [16]. During a long-duration bed rest, bone resorption markers were increased, and oxidative damage markers were increased. Following 90 d of bed rest, total antioxidant capacity was decreased. Also, iron status indices exhibited patterns of elevated iron stores with a reduced concentration of transferrin receptors [16]. Xu et al. [44] observed that HLU-induced bone loss in mice was related to iron overload, and coupled with the hepcidin, which is the main regulator of iron homeostasis secreted from the liver. Recently, we also found a similar result from mice after HLU for 28 days, and we proposed a hypothesis that high levels of oxidative stress caused by iron overload is a trigger of unloading-induced bone loss [18]. Indeed, unloaded mice showed significantly increased intracellular ROS and antioxidant enzyme SOD1 (CuZnSOD) in in bone and bone marrow cells [19]. Furthermore, SOD1 deficiency enhanced bone loss by decreased osteoblastic abilities during mechanical unloading, whereas the treatment of an antioxidant, vitamin C, significantly attenuated unloading-induced bone loss [19]. Curcumin or hydrogen molecule alleviated HLU-induced bone loss in rats, possibly through abating oxidative stress markers such as MDA and increasing antioxidant markers such as total sulfhydryls in femurs [90,91]. Osteoblastic MC3T3-E1 cells that were cultured in a rotary wall vessel bioreactor (RWVB, a facility for simulating microgravity) had higher ROS levels, but lower differential abilities. Conversely, RWVB exposure induced the increase in ROS production and promoted the osteoclastogenesis of RAW264.7 cells [90,91]. These findings elucidated that the increase of iron stores and ROS production in response to microgravity, damaged the normal function of osteoblasts, but promoted osteoclastic capability, which resulted in bone disorder.

In addition to microgravity, space-like radiation-induced bone loss was documented in many studies [92], and the possible mechanisms are associated with altered iron metabolism. Budagov et al. [93] reported that the iron content in blood reflects the severity of radiation damage. Elevated ferric ions in mice serum were found under high doses of gamma radiation [94,95]. Iron chelators, such as deferoxamine (DFO), can ameliorate the negative effects of radiation on bone. DFO treatment markedly alleviated the reduced bone mass and mechanical properties induced by radiation [96]. Significant increases in the metrics of callus size, mineralization, and strength of DFO-treated mandibles were compared with the irradiated fracture [97]. The increase of ROS and the decrease of anti-oxidative enzymes are another possible mechanism for radiation-induced bone damage. Irradiation suppressed osteogenic differentiation of MC3T3-E1 cells, and promoted the expression of Nrf2 and heme oxygenase-1 (HO-1); the accumulation of cellular oxidants and the consumption of antioxidant defense enzymes [98]. Radiation also inhibited osteoblast differentiation and induced ROS production in bone marrow-derived skeletal cell progenitors in a dose-dependent manner [99]. Reduced cancellous bone volume fractions in the proximal tibia and lumbar vertebrae, and elevated the osteoclast surface in the tibia, and ROS production in marrow cells were observed in male C57BL/6 mice after the exposure of gamma irradiation [79]. Treatment with exogenous SOD could protect marrow-derived osteoprogenitors from the adverse effects of exposure to low-linear-energy-transfer (LET) protons in vitro [100].

### 4.2. Iron and ROS Signaling in Spaceflight Induced-Muscle Atrophy

Muscle atrophy due to disuse is a serious issue for humans in spaceflight and immobilized patients on Earth. It is well known that gravitational unloading of human and mammalian skeletal muscles can cause muscular atrophy in spaceflight, including changes in muscle fiber composition, gene expression, and a decrease in regenerative muscle growth [5,101]. Various models of muscular unloading, including bed rest, HLU, immobilization and reduced step, were used in laboratory experiments, and have provided specific fundamental information concerning the morphological, biochemical, and molecular processes that induce the degeneration and atrophy of muscle fibers [102]. Briefly, the current understanding of muscular disuse atrophy is that the physical signals of mechanical unloading is transformed into a molecular signaling process that induces the degradation of myofibrils at a biochemical level, followed by a significant degree of muscle mass loss [103]. In addition, the possible mechanism processes are concerned with altered iron metabolism and oxidative damage in humans/animals undergoing mechanical unloading has also been reported.

Iron catalyzes the production of ROS. Excess levels of iron and ROS are considered to result in the fatigue of skeletal muscle and the loss of skeletal muscle mass with age [20,104]. A mouse model of iron overload showed less work in the endurance test, producing less force in the skeletal muscle strength test. Concomitantly, on the one hand, the levels of iron and ferritin were increased in the tibialis anterior muscle; on the other hand, the oxidative stress product MDA and the antioxidant enzyme activity of GSH reductase and GSH peroxidase were elevated in the iron group, compared with the control group [11]. The iron-injected mice displayed an increase of iron levels and ferritin content in the skeletal muscle and serum, along with reduced skeletal muscle mass, which may have been induced by decreasing the phosphorylation of Akt and forkhead box O3 (FOXO3a) in skeletal muscles [105]. Furthermore, treatment by antioxidant could abrogate iron loading-induced muscle atrophy by activating the Akt-FOXO3a pathway [105]. In vitro, Kasztura et al. [106] used ferric ammonium citrate (FAC) to treat rat skeletal myocyte L6G8C5 cells, which showed reduced myoglobin levels and decreased proliferative activity in an iron overload condition. In hemodialysis patients, increased serum ferritin significantly correlated with handgrip strength and muscle quality, and thus suggested that iron overload should be concerned, to avoid its possibly detrimental effect on muscle in such patients [107].

Long-term bed rest induces muscle atrophy and reduced muscle strength. Changes in muscle size and strength of the antigravity muscles (soleus, gastrocnemius, and vastus lateralis) have been studied after bed rest in various durations [102]. Meanwhile, the enhancement of oxidative damage involved in muscle atrophy caused by bed rest. After 35 days of bed rest, but not eight days, the vastus lateralis muscle displayed muscle atrophy (18%) and elevated protein carbonylation (oxidative damage of protein) [17,108]. On Day 8, the transient increase in two heat shock proteins, heme oxygenase-1 (HO-1) and mitochondrial Hsp-70 isoform (Grp75) [17], was found to be up-regulated in response to ROS production [109]. A global analysis of gene expression patterns in sedentary men revealed that the pathways involved in oxidative stress were up-regulated following 48 hr of unloading via unilateral lower limb suspension (ULLS) [110]. Recently, a report compared two human disuse models of bed rest and ULLS [111]. The loss of muscle fiber cross-sectional area and myosin concentration was very similar in both disuse models. However, although all antioxidant enzymes were down-regulated and protein carbonylation was enhanced in bed rest, antioxidant defense systems were up-regulated, and no protein carbonylation occurred in ULLS [111].

In all models of muscle atrophy caused by microgravity, HLU and immobilization are the most widely methods for studying skeletal muscle atrophy in small mammals. An imbalance of iron and redox homeostasis can be found in atrophic muscle induced by HLU. Following 14 days of HLU in 32-month-old male Fischer 344/Brown Norway rats, demonstrated that oxidative damage of RNA increased by 36%, and non-heme iron levels were elevated by 83%, with HLU in the gastrocnemius muscle [20]. The positive association between iron levels and oxidative stress markers supports the viewpoint that higher levels of non-heme may be associated with the release of more free iron, which has a strong catalytic potential for cellular damage. In another similar study, the effects of seven days and 14 days of reloading after 14 days of HLU on iron metabolism and oxidative injury in the gastrocnemius muscle of rats were investigated [112]. The results showed that the muscle mass was decreased, and the levels of non-heme iron and oxidative damage was increased in the gastrocnemius muscle of old rats after unloading for 14 days. Skeletal muscle of aged rats did not recover from muscle atrophy in seven and 14 days of reloading. Although there were no changes in elevated levels of non-heme iron, oxidative damage was decreased after reloading for seven and 14 days [112]. Mice were subjected to HLU for two weeks; the results suggested that imbalanced redox homeostasis occurred during muscle atrophy, due to the activation of NOX1, the deficiency of mitochondrial complex I, and the interference of antioxidants [113]. It has been well demonstrated that reduced protein synthesis and elevated protein degradation in muscle seems to be the major trigger in disuse muscle atrophy. Oxidative stress has been recognized to retard the synthesis of protein and motivate protein degradation in disused muscle [114]. A decrease in Akt phosphorylation often leads to higher protein degradation and decreased synthesis rates. Investigations showed that the increase in mitochondrial-derived ROS was caused by disuse-induced attenuation in Akt activity [115]. Elevated oxidants in the immobilized soleus induced significant reductions in Akt phosphorylation. Moreover, increased oxidative stress attributed to hindlimb immobilization was shown to markedly elevate the expression of FoxO3a target genes, including MuRF1 and MAFbx/Atrogin 1, which led to atrophy [116]. Indeed, disuse-associated oxidants has been shown to result in an increase in dephosphorylation of cytoplasmic FoxO3a [116]. Additionally, increased levels of oxidative stress induced by disuse can facilitate proteolysis in muscle fibers through oxidizing proteins, thus enhancing their sensitivity to proteolytic degradation. For instance, antioxidant supplementation during HLU effectively decreases the ubiquitination and fragmentation of myosin heavy chain proteins [117]. In short, the results of these study strongly suggest a risky role of oxidative stress in the regulation of muscle protein degradation, and synthesis in HLU and immobilization.

## 5. Implications for the Development of Spaceflight Countermeasures

According to the above review, it is necessary to develop effective countermeasures against iron overload and oxidative damage in bone and muscle during spaceflight. Greater investment in ground-based or spaceflight studies showed some methods against iron overload and oxidative damage induced by microgravity and space radiation.

At present, iron chelation therapy is the most effective treatment for systemic iron overload. At present, three main iron chelators, including DFO, deferiprone, and deferasirox, are used in clinical practice [118]. Using iron chelators to reduce iron is worth investigating for the attenuation of bone loss and muscle atrophy in the space environment. The iron chelator DFO decreased iron levels in the liver and hindlimb bones, and ameliorated unloading-induced bone loss through upregulating osteoblast activity markers, downregulating osteoclast activity genes, and improving trabecular architecture [44]. DFO treatment notably reduced the decreased bone mass and the mechanical properties induced by radiation [96,119]. Observations indicated that eliminating iron using an iron chelator, DFO, suppressed increased oxidative stress and ameliorated muscle atrophy caused by immobilization in young rats [120]. This result indicates that iron plays a very important role in enhancing the oxidative stress of atrophied muscle, and that DFO can decrease the degree of atrophy by suppressing oxidative stress. Consequently, iron chelation therapy may be engaged in the prevention or management of spaceflight-related bone loss and muscle atrophy, although the number of studies specializing in this issue are still limited.

Exposure to the space environment usually produces a large amount of ROS, which then elevates oxidative stress and finally results in bone and muscle damage. Thus, some agents with antioxidant properties can mitigate microgravity and cosmic-related radiation induced bone loss and muscle atrophy. Allopurinol is a xanthine oxidase inhibitor with antioxidant properties, and it has been shown to protect the function of unloaded antigravity muscles [121]. Data from a study indicated that HLU caused an oxidative response in rats, and this response disappeared in the presence of L-carnitine, which is a well-known intensifier of the mitochondrial antioxidant system activity [72]. Aminoguanidine is a nucleophilic hydralazine compound that is an antioxidant against ROS and lipid peroxidation, and has also been shown to reduce HLU-induced oxidative stress [71]. Treatment of γ-irradiated rats with the antioxidant, N-acetylcysteine (NAC), exhibited a significant reduce in the levels of MDA, nitrate/nitrite, and DNA damage [122]. In addition, NAC could prevent the development of trabecular bone abnormalities in mice with iron overload [8]. Serum and salivary vitamin C and E concentrations were significantly decreased in the microgravity environment [62]. Accordingly, supplementation of antioxidant vitamin C or E exhibited an effective protection for bones under microgravity [19,123]. Vitamin E supplementation could also prevent unloading-induced atrophy in the soleus muscle [124].

Numerous natural products have displayed benefits against oxidative stress in bones and muscles. Curcumin, a phenolic natural product, is extracted from the rhizome of *Curcuma longa*. Curcumin alleviated HLU-induced bone loss and oxidative stress by reducing the MDA content and elevating the total sulfhydryl content in femurs [90]. In vitro, curcumin inhibited simulated microgravity-induced ROS production and increased osteoblastic differentiation in MC3T3-E1 cells. On the contrary, curcumin decreased mimical microgravity-induced ROS production and reduced osteoclastogenesis [90]. Moreover, curcumin had a preventive effect on peroxidative damage in bed rest [63]. Tanshinol, isolated from *Salvia miltiorrhiza*, rescued the retardation of osteoblastic differentiation through suppressing the activation of the FoxO3a transcription factor and counteracting the inhibition of Wnt signaling under the condition of oxidative stress [125]. Salvianolic acid B is a water-soluble substance of *Salvia miltiorrhiza*, and it has a protective effect on osteoblasts, through the stimulation of osteoblast activity and antioxidant ability under simulated microgravity [126]. Some flavonoids, such as isorhamnetin and luteolin, could prevent microgravity-caused oxidative stress in neuroblastoma SH-SY5Y cells, through restraining the ROS-NO pathway [127]. Flavonoid quercetin was found to have antioxidant properties, and it can prevent muscle atrophy through a decrease in the expression of atrogin-1/MuRF-1 in HLU mice [128]. Resveratrol, a polyphenol in red wine that is currently being studied extensively due to its antioxidant activity, has been shown to prevent iron overload induced bone loss [129]. Prior treatment with resveratrol significantly prevented bone loss caused by hindlimb immobilization in rats [130]. Likewise, resveratrol treatment maintained muscle mass and the maximal muscular contraction force in the soleus of rats subjected to HLU for 15 days, and also entirely maintained the ability of soleus mitochondria oxidization of palmitoyl-carnitine, and inverted the reduction in the ratio of glutathione to glutathione disulfide, which is a biomarker of oxidative stress [131]. Dietary supplement of a soy protein extract, the Bowman–Birk inhibitor concentrate (BBIC) could protect the skeletal muscle of mice during HLU, and it promoted redox homeostasis in muscle fibers [132]. Dried plum (DP) was previously shown to have an excellent antioxidant activity [133]. DP diet was most effective in preventing radiation-induced increases in bone resorption-related gene expression and ensuing cancellous bone loss, compared with other antioxidants (antioxidant cocktail, dihydrolipoic acid, and ibuprofen) [134]. Hydrogen was recently found to be a new type of therapeutic medical gas in various biomedical fields, as it has amazing antioxidant properties [135]. Treatment with hydrogen water ameliorated the HLU-induced reduction of BMD, stiffness, ultimate load and energy, simultaneously alleviated the HLU-induced enhancement of MDA and peroxynitrite levels, and abated the HLU-induced reduction of total sulfhydryl levels in the femur and lumbar vertebra [91]. Furthermore, it was hypothesized that treating astronauts by inhaling or drinking hydrogen water (HW) may be a new strategy to prevent and treat radiation-induced oxidative injuries during space missions [136]. Even though these data are all from ground models, they can still provide enlightenment for the development of countermeasures against bone loss caused by oxidative stress during spaceflight.

Establishing a suitable dietary system is necessary for humans in long-term spaceflight. It seems that intake of iron chelators or/and rich antioxidants will avert iron overload and oxidative damage induced by the space environment. More studies are warranted to select optimal iron chelators and antioxidants, to prevent physiological damage caused by space travel.

## 6. Concluding Statements

**Figure 1:**
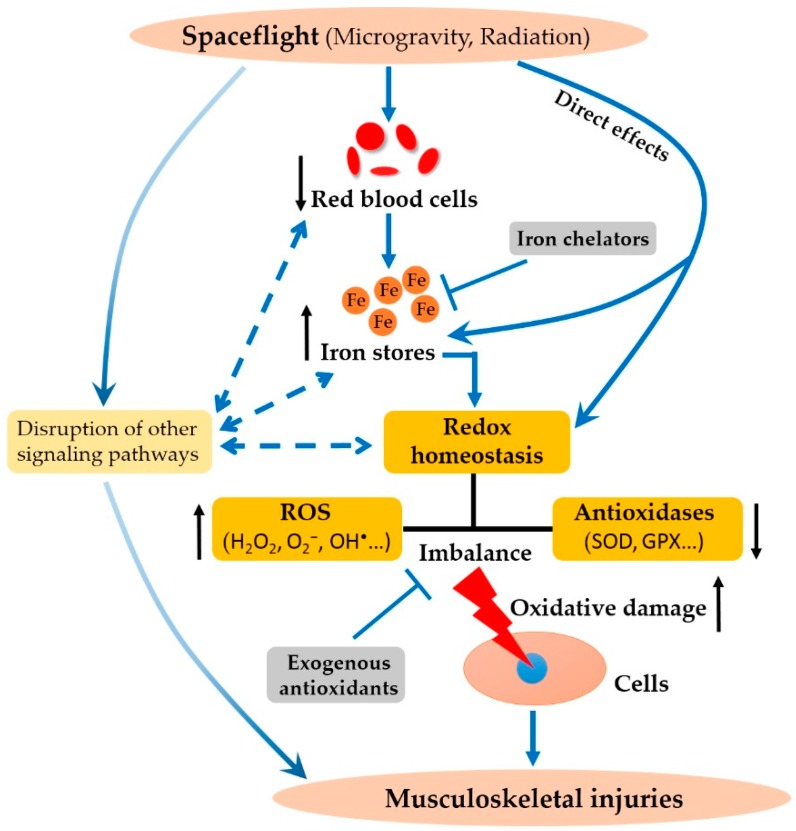
Hypothetical model on how spaceflight leads to injuries in the musculoskeletal system. Exposure of tissues to the spaceflight environment, such as microgravity and radiation, leads to a decreased mass of red blood cells. This induces elevated iron stores, due to iron release from destroyed erythrocytes. Excess iron leads to an imbalance in redox homeostasis, owing to increased levels of reactive oxygen species (ROS) and reduced levels of antioxidases. The spaceflight environment may also have direct effects on iron metabolism and redox homeostasis. The imbalance of redox status leads to the oxidative damage of cells, which in turn result in injuries in the musculoskeletal system. Other signaling processes are disrupted by the spaceflight environment, and may also cause these defects. Some exogenous iron chelators and antioxidants may block the increases in iron and the imbalance in redox status, thereby preventing oxidative damage.

Microgravity and cosmic radiation are the main risk factors that threaten the survival of living organisms. Iron overload and oxidative stress triggered by spaceflight induces a variety of damage to the human body, including the degradation of bone and muscle. In this review, we demonstrated a connection between iron overload, oxidative damage, and musculoskeletal injury, which arise from exposure to the space environment (Figure 1). We discussed the potential mechanisms by which the spaceflight environment induces deterioration in the musculoskeletal system. Herein, we believe that increased iron stores and impaired redox homeostasis are two important inducing factors. Some countermeasures that have therapeutic potential for the loss of bone and muscle through alleviating the levels of iron stores and oxidative stress, are also listed in this review.

Although some progress has been made in studies related to the spaceflight environment, some problems remain unresolved. It is well known that bone marrow is essential for hematopoiesis, and numerous studies have shown that deterioration of bone metabolism results in changes in the bone marrow microenvironment, thereby affecting hematopoietic function [137]. Moreover, rich iron is present in muscle fibers (in myoglobin); thus, the body’s iron stores may be affected the loss of muscle under microgravity. Therefore, a prominent problem that should be addressed is whether iron loading induces or results from the progressive deterioration of the musculoskeletal system under the spaceflight environment. More studies should be taken to thoroughly illustrate the causal relationship and time series between iron loading and musculoskeletal deterioration. By taking samples at different times, using genetic models and isotope-labeled iron will contribute to resolving this question. However, we should understand that the chances of real spaceflight are limited, and the sample types from astronauts are restricted and the subject pools are small. Consequently, some ground-based models can be used as crucial experimental platforms to allow researchers to examine the effects of the space environment on the musculoskeletal system.
